# Chronic Stress Oppositely Regulates Tonic Inhibition in Thy1-Expressing and Non-expressing Neurons in Amygdala

**DOI:** 10.3389/fnins.2020.00299

**Published:** 2020-04-17

**Authors:** Han-Qing Pan, Wen-Hua Zhang, Cai-Zhi Liao, Ye He, Zhi-Ming Xiao, Xia Qin, Wei-Zhu Liu, Na Wang, Jia-Xin Zou, Xiao-Xuan Liu, Bing-Xing Pan

**Affiliations:** ^1^Department of Biological Science, School of Life Sciences, Nanchang University, Nanchang, China; ^2^Laboratory of Fear and Anxiety Disorders, Institutes of Life Science, Nanchang University, Nanchang, China; ^3^Center for Medical Experiments, Nanchang University, Nanchang, China; ^4^Department of Physiology, Mudanjiang Medical University, Mudanjiang, China

**Keywords:** chronic stress, amygdala, tonic inhibition, Thy1, neuronal excitability, corticosterone

## Abstract

Chronic or prolonged exposure to stress ranks among the most important socioenvironmental factors contributing to the development of neuropsychiatric diseases, a process generally associated with loss of inhibitory tone in amygdala. Recent studies have identified distinct neuronal circuits within the basolateral amygdala (BLA) engaged in different emotional processes. However, the potential circuit involved in stress-induced dysregulation of inhibitory tones in BLA remains elusive. Here, a transgenic mouse model expressing yellow fluorescent protein under control of the Thy1 promoter was used to differentiate subpopulations of projection neurons (PNs) within the BLA. We observed that the tonic inhibition in amygdala neurons expressing and not expressing Thy1 (Thy1+/−) was oppositely regulated by chronic social defeat stress (CSDS). In unstressed control mice, the tonic inhibitory currents were significantly stronger in Thy1- PNs than their Thy1+ counterparts. CSDS markedly reduced the currents in Thy1- projection neurons (PNs), but increased that in Thy1+ ones. By contrast, CSDS failed to affect both the phasic A-type γ-aminobutyric acid receptor (GABA_A_R) currents and GABA_B_R currents in these two PN populations. Moreover, chronic corticosterone administration was sufficient to mimic the effect of CSDS on the tonic inhibition of Thy1+ and Thy1- PNs. As a consequence, the suppression of tonic GABA_A_R currents on the excitability of Thy1- PNs was weakened by CSDS, but enhanced in Thy1+ PNs. The differential regulation of chronic stress on the tonic inhibition in Thy1+ and Thy1- neurons may orchestrate cell-specific adaptation of amygdala neurons to chronic stress.

## Introduction

Exposure to extreme or prolonged stress leads to a spectrum of brain and behavior abnormalities and is associated with the onset and exacerbation of various neuropsychiatric diseases including anxiety disorder and depression (de Kloet et al., 2005; McEwen, 2007). Amygdala, a diamond-shaped region located deep inside the temporal lobe, is critically engaged in encoding external information with distinct valence (Janak and Tye, 2015). Accumulating evidence in past decades has identified amygdala as a primary target of stress and a key mediator of stress adversity in brain. For example, chronic stress causes considerable structural and functional remodeling of amygdala neurons (Vyas et al., 2002; Zhang J.Y. et al., 2019), resulting in persistent and excessive activation of amygdala (Rosenkranz et al., 2010; Zhang W.H. et al., 2019) and contributing to pathogenesis of stress-related mental disorders (Qin et al., 2015; Zhou et al., 2019).

Under resting state, amygdala has a highly inhibitory tone, which distinguishes it from its proximal regions (LeDoux, 2007). This provides a brake for amygdala to limit its activation by the neutral information in the environment, thus avoiding excessive or inappropriate expression of emotion (Quirk and Gehlert, 2003; Jie et al., 2018). However, under some adverse conditions such as prolonged exposure to stressful events, the high inhibitory tone is removed, leading to persistent and excessive activation of amygdala (Manzanares et al., 2005; Liu et al., 2014). We have recently observed that the impaired inhibition in amygdala by chronic stress is primarily due to the loss of tonic inhibition (Liu et al., 2014), which is mediated by extrasynaptic GABA_A_Rs, rather than the phasic inhibition by synaptic GABA_A_Rs. Notably, both preclinical and clinical evidence has demonstrated that GABAergic disinhibition is highly linked to the pathogenesis of multiple neuropsychiatric diseases (Brambilla et al., 2003; Bohnsack et al., 2018; Zhang et al., 2018) and the therapeutic effect of many anxiolytic agents acts at least partially through augmenting GABAergic inhibition in amygdala (Nuss, 2015). Thus, understanding the precise mechanisms underlying stress-induced GABAergic disinhibition in amygdala may yield novel targets for prevention and treatment of stress-related neuropsychiatric disorders.

In recent years, evidence has been accumulated to dissect the structure and function of individual amygdala neurons at the molecular and circuit levels (Senn et al., 2014; Kim et al., 2016). Thy1 is a glycophosphatidylinositol-anchored glycoprotein expressed on the cell membrane of various types of cells (Williams and Gagnon, 1982). Interestingly, Thy1 has been recently used as a neuronal marker to differentiate subpopulations of the projection neurons (PNs) in the basolateral amygdala (BLA) express Thy1, and Thy1+ and Thy1- PNs appear to be differently engaged in amygdala-related tasks (Jasnow et al., 2013; McCullough et al., 2016; Gilman et al., 2018). For example, the Thy1+ PNs in amygdala were activated during fear extinction and extinction retention, whereas the Thy1- neurons were recruited during fear expression (McCullough et al., 2016). Nonetheless, how chronic stress impacts the GABAergic signal in the two populations in amygdala is yet unknown.

To answer this question, we subjected the mice with the expression of YFP driven by the Thy1 promoter to chronic social defeat stress (CSDS) from postnatal day 35 through day 45. We observed that CSDS markedly reduced the tonic inhibitory currents in Thy1- PNs but increased those in Thy1+ PNs, with negligible influence on the phasic GABA_A_R currents and GABA_B_R-mediated currents in both neuronal populations. The opposite influences of CSDS could be readily mimicked by chronic administration of corticosterone (CORT). As a consequence, the GABAergic suppression of the excitability of Thy1- and Thy1+ PNs was weakened and augmented by CSDS, respectively.

## Materials and Methods

### Animals

Male B6.Cg-Tg (Thy1–YFP) HJrs/J (Thy1–YFP) mice and CD-1 retired breeders were used in this study. All animals were housed in a temperature-maintained, humidity-controlled colony with convenient access to food and water, and a 12:12 h/light–dark cycle (7 a.m. to 7 p.m.). All experimental procedures were approved by the Institutional Animal Care and Use Committee of Nanchang University.

### Chronic Social Defeat Stress

The CSDS was performed as described previously (Golden et al., 2011). Briefly, the Thy1–YFP intruder mouse was exposed to a different aggressive resident CD-1 mouse for 5 min each day and lasted for 10 consecutive days. After 5 min of social defeat, the intruder mouse and the resident mouse were separated by a perforated acrylic plate for 24 h to maintain a sensory stress to the intruder mice. The control mice were housed in equivalent cages but with members of the same strain, which were changed daily.

### Chronic Corticosterone Administration

Corticosterone was dissolved in EtOH as the stock solution and freshly prepared in drinking water every day (EtOH < 1‰ at final concentration) and delivered in bottles, which were wrapped with silver paper to prevent from light. The vehicle-treated mice were allowed to drink water containing 1‰ ethanol. The entire procedure lasted for 10 days.

### Open Field Test

Open field test (OFT; Med Associates Inc., Fairfax, VT, United States) was performed as previously described (Zhang W.H. et al., 2019). Briefly, mice were habituated in the test room for 30 min, followed by placing mice in a square chamber (50 × 50 cm) with white light. The mouse activity was monitored for 10 min. The center square (25 × 25 cm) of the chamber was defined as the center area, and the time spent in the center area and entries in the center area were assessed by the video monitoring software (SOF-842; Med Associates Inc.).

### Elevated Plus Maze Test

Elevated plus maze (EPM) test was performed as previously prescribed (Zhang W.H. et al., 2019). Briefly, after a 30 min habituation in the behavior test room with red light, experimental mice were mildly placed in the hub area facing one of the closed arms of EPM apparatus (Med Associates Inc., St. Albans, VT, United States). Their behaviors were recorded for 10 min by the video tracking software (DOC-086; Med Associates Inc.). Anxiety-like behavior was measured by the time spent and the number of entries in open arms.

### Immunohistochemistry

The mice were anesthetized by isoflurane, followed by perfusion with phosphate-buffered saline (PBS) and 4% paraformaldehyde (PFA) successively to fix the tissues. The brains were then postfixed in 4% PFA for 24 h. Subsequently, the brains were moved to the chamber containing PBS, and 40 μm slices including amygdala were sectioned by tissue slicer (VT 1000S Vibratome; Leica Microsystems, Wetzlar, Germany) and stored in Section Storage Solution (FD NeuroTechologies Inc., Columbia, MD, United States).

The brain slices were rinsed in PBS (4 × 10 min) and then transferred to blocking buffer (PBS with 0.1% Triton X-100 containing 10% donkey serum) for 2 h at room temperature. Then, the slices were incubated overnight at 4°C with primer antibody against CaMKIIα (goat polyclonal antibody, 1:500; Abcam, Cambridge, United Kingdom); after that, three times rinses in PBS with 0.1% Triton X-100 before incubation with second antibody at room temperature for 2 h were conducted. Subsequent to three times washes, the slices were paved on the microslides and mounted with Fluoromount Aqueous Mounting Medium (Sigma-Aldrich, St. Louis, MO, United States). The images were taken by a confocal laser-scanning microscopy (Olympus FV1000; Olympus Corp., Tokyo, Japan) and processed by Olympus Fluoview (Olympus Corp.).

### Electrophysiological Recording

The *in vitro* electrophysiological recordings were performed as previously described (Liu et al., 2014). Briefly, 1 day after the last episode of CSDS or chronic corticosterone administration, the mice were anesthetized with isoflurane and then sacrificed by decapitation. Brains were then removed immediately to ice-cold oxygenated (95% O_2_/5% CO_2_) partial sucrose artificial cerebrospinal fluid (ACSF) containing 80 mM NaCl, 3.5 mM KCl, 4.5 mM MgSO_4_, 0.5 mM CaCl_2_, 1.25 mM NaH_2_PO_4_⋅2H_2_O, 25 mM NaHCO_3_, 10 mM glucose, and 90 mM sucrose (pH, 7.3–7.4). The brain slices containing BLA of 320 μm were cut and collected by using the tissue slicer (VT 1000S Vibratome; Leica Microsystems) mentioned previously (Song et al., 2017) and then transferred to preheated normal ACSF containing 124 mM NaCl, 2.5 mM KCl, 1 mM MgSO_4_, 2.5 mM CaCl_2_, 10 mM glucose, and 22 mM NaHCO_3_ at 35°C for 30 min. After that, the slices were maintained at room temperature at least 1 h before recording. Slices were moved to the recording chamber and perfused with ACSF at the rate of 2 mL/min. The temperature was kept at 30°C ± 1°C using temperature controller (TC-324B; Warner Instrument Co., Hamden, CT, United States). Whole-cell patch clamp was performed in all electrophysiological recordings. Signals were amplified and digitized by using Axon 700B Amplifier and Digidata 1440A (Molecular Device, San Jose, CA, United States) digital analog convertor, respectively. To record GABAergic signal, the recording pipettes were prepared by Sutter P97 horizontal puller and filled with 100 mM CsCl, 30 mM Cs-methane sulphonate, 5 mM KCl, 2 mM MgCl_2_, 10 mM HEPES, 0.2 mM EGTA, 2 mM ATP-Mg, and 0.1 mM GTP-Na; the pH was adjusted to 7.3–7.4 with CsOH and osmolarity to 290 mOsm with sucrose. To record GABA_B_R currents and neuronal excitability, the pipette solution was replaced by those containing 130 mM K-gluconate, 5 mM KCl, 2 mM MgCl_2_, 10 mM HEPES, 0.2 mM EGTA, 2 mM ATP-Mg, and 0.1 mM GTP-Na, adjusted the pH to 7.3–7.4 with KOH and the osmolarity to 290 mOsm with sucrose. To record phasic and tonic GABA_A_R currents, the membrane potential was held at -70 mV and 20 μM CNQX; 50 μM DL-APV and 5 μM CGP52432 were routinely applied in bath solution to block AMPAR, NMDAR, and GABA_B_R currents, respectively. After the spontaneous inhibitory postsynaptic currents (sIPSCs) were collected for 2–3 min, 5 μM THIP or GABA was added to ensure the activation of extrasynaptic GABA_A_R, followed by 100 μM picrotoxin (PTX) to block GABA_A_R currents. To record GABA_B_R currents, cells were held at -50 mV and 20 μM CNQX; 50 μM DL-APV and 100 μM PTX were included in ACSF to prevent the interference from the currents induced by ionotropic glutamate receptors and GABA_A_Rs. To activate GABA_B_R, 10 μM baclofen was added followed by 5 μM CGP52432 to block GABA_B_Rs. In experiments recording neuronal excitability, the current clamp mode was performed, and the pulsed depolarization currents with increasing amplitude at step of 50 pA (Zhang W.H. et al., 2019) or ramped depolarization current (Qin et al., 2019; Jin et al., 2020) were injected. The pipette resistance was 3–7 MΩ. Series resistance (Rs) was in the range of 10–20 MΩ and monitored throughout the experiments. If Rs changed more than 20% during recording, the data would be excluded. Data were low-pass filtered at 3 kHz and digitized at 10 kHz.

### Statistics

**FIGURE 1:**
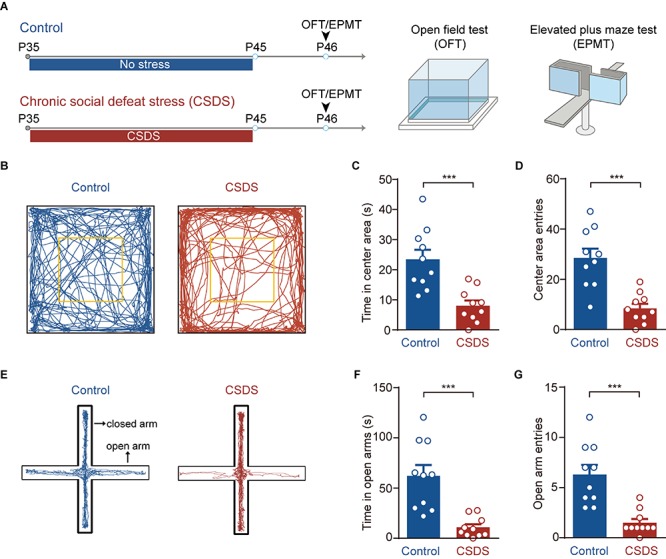
Chronic social defeat stress (CSDS) increases anxiety-like behavior in mice. **(A)** Schematic showing procedures for unstressed control mice and CSDS mice. Anxiety-like behavior was measured by open field test (OFT) and elevated plus maze (EPM). **(B)** Representative activity tracking in the OFT in control and CSDS mice. **(C)** Time in center area. **(D)** Center area entries. **(E)** Representative activity tracking in the EPM in control and CSDS mice. **(F)** Elevated plus maze test open arm time. **(G)** Elevated plus maze test open arm entries. ***p < 0.001. Pooled data are presented as mean ± SEM.

**FIGURE 2:**
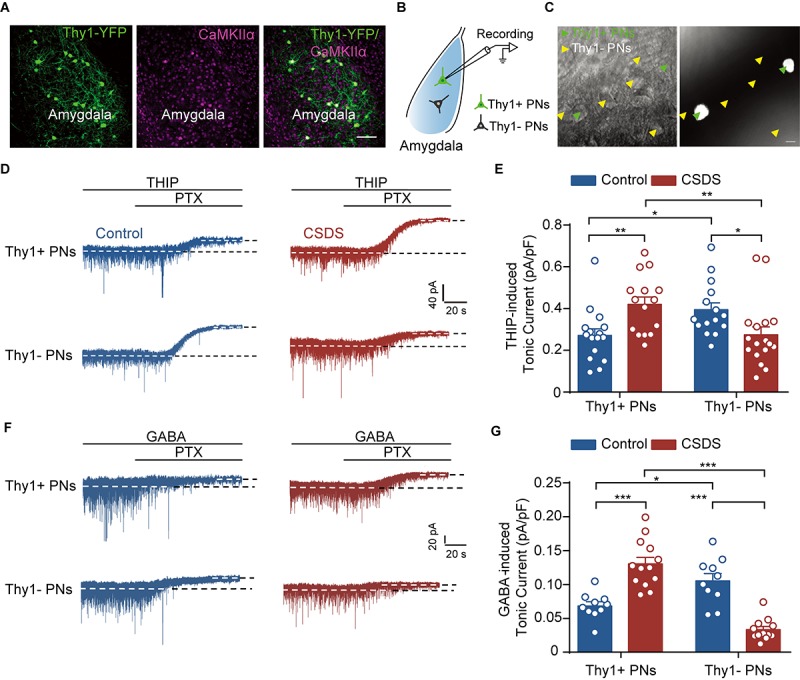
Chronic social defeat stress increases tonic GABA_A_R currents in Thy1+ neurons, but reduces those in Thy1- neurons, in BLA. **(A)** Representative image showing the expression of Thy1–YFP in amygdala. CaMKIIα was used as a marker for projection neurons (PNs). The scale bar represents 100 μm. **(B)** Schematic showing whole-cell patch clamp recording of PNs in amygdala. **(C)** Infrared DIC (left) or fluorescent (right) images of Thy1+ neurons (green arrow heads) and Thy1- neurons (yellow arrowheads). The scale bar represents 15 μm. **(D)** Representative traces of THIP-induced tonic GABA_A_R currents recorded in Thy1+ and Thy1- neurons from control and CSDS mice. **(E)** Average of THIP-induced tonic GABA_A_R currents between Thy1+ neurons and Thy1- neurons. **(F)** Representative traces showing GABA-induced tonic GABA_A_R currents recorded in Thy1+ and Thy1- neurons from control and CSDS mice. **(G)** Comparison of GABA-induced tonic GABA_A_R currents in Thy1+ and Thy1- neurons. *p < 0.05, **p < 0.01, ***p < 0.001. Pooled data are presented as mean ± SEM.

**FIGURE 3:**
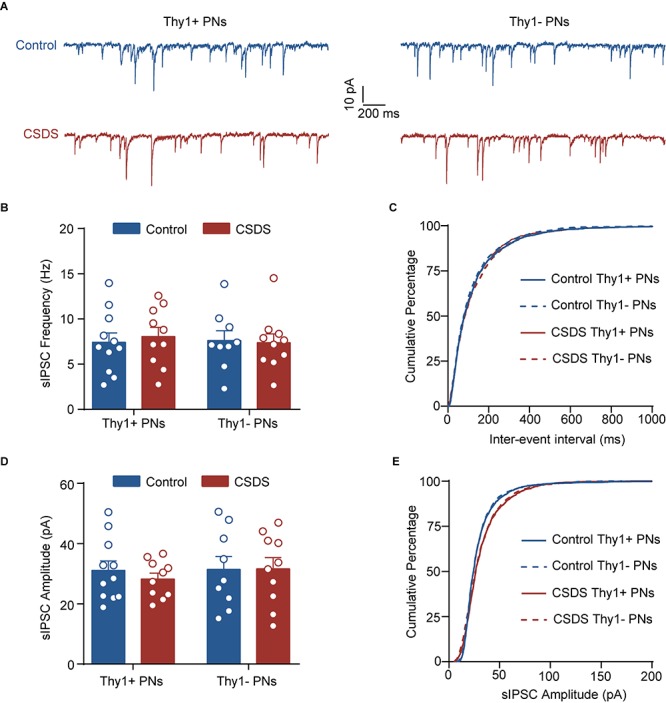
Chronic social defeat stress has little effect on phasic GABA_A_R current in either Thy1+ or Thy1- neurons in BLA. **(A)** Representative traces of spontaneous inhibitory postsynaptic current (sIPSC) in Thy1+ and Thy1- neurons from control and CSDS mice. **(B)** Average of sIPSC frequency. **(C)** Cumulative probability of interevent interval of sIPSC. **(D)** Average of sIPSC amplitude. **(E)** Cumulative probability of amplitude of sIPSC. Pooled data are presented as mean ± SEM.

**FIGURE 4:**
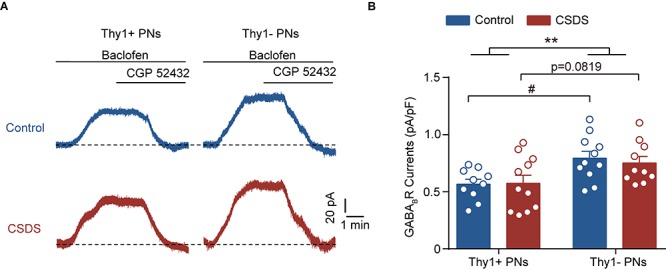
Chronic social defeat stress has no effect on the GABA_B_R currents in either Thy1+ or Thy1- neurons. **(A)** Representative traces showing baclofen-induced GABA_B_R currents in both neuron types. **(B)** Summary plot of amplitude of GABA_B_R currents. **p < 0.01 vs. Thy1+ group/Thy1- group, ^#^*p* < 0.05 vs. Thy1+ /Thy1- neurons in control mice. Pooled data are presented as mean ± SEM.

**FIGURE 5:**
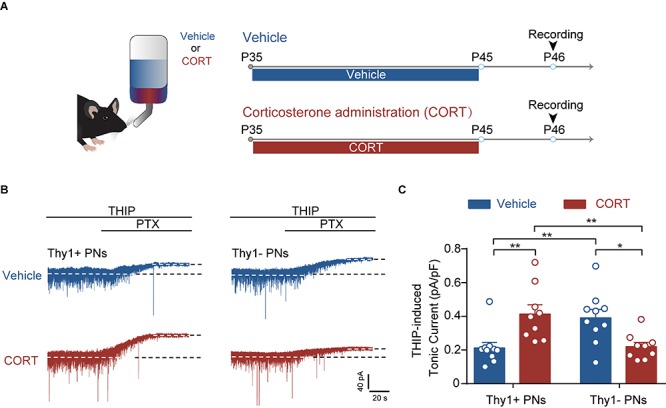
Chronic corticosterone administration mimics the effect of CSDS on tonic inhibition in Thy1+ and Thy1- neurons in BLA. **(A)** Schematic showing procedures for vehicle mice and chronic corticosterone administration (CORT) mice. **(B)** Representative traces of THIP-induced GABA_A_R currents of Thy1+ and Thy1- neurons from vehicle- and CORT-treated mice. **(C)** Average of THIP-induced GABA_A_R currents in Thy1+ and Thy1- neurons. *p < 0.05, **p < 0.01. Pooled data are presented as mean ± SEM.

**FIGURE 6:**
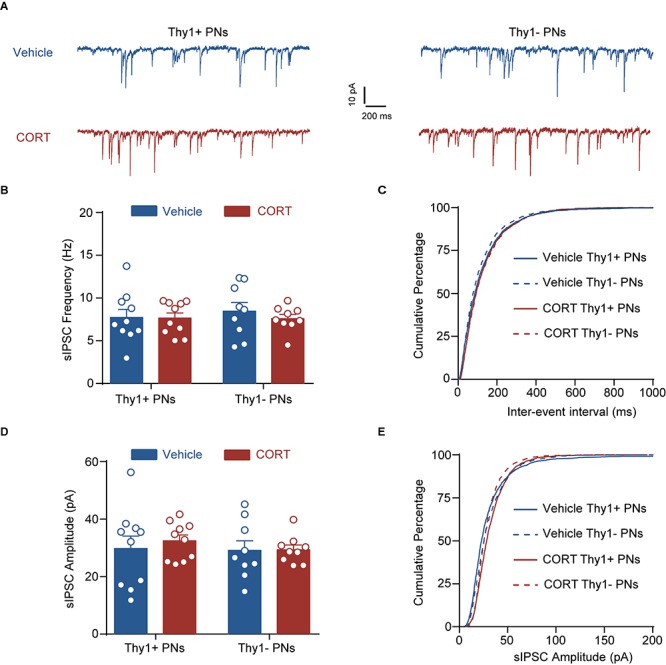
Chronic corticosterone administration has no effect on the phasic inhibition in Thy1+ and Thy1- neurons. **(A)** Representative traces of spontaneous inhibitory postsynaptic current (sIPSC) in Thy1+ and Thy1- neurons from vehicle and CORT mice. **(B)** Average of sIPSC frequency. **(C)** Cumulative probability of interevent interval of sIPSC. **(D)** Average of sIPSC amplitude. **(E)** Cumulative probability of amplitude of sIPSC.

**FIGURE 7:**
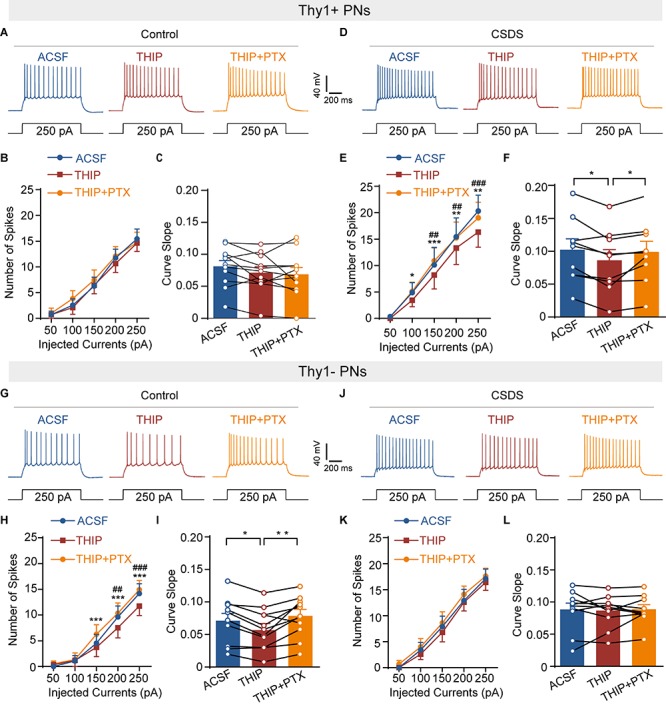
Chronic social defeat stress increases GABAergic modulation of neuronal excitability of Thy1+ neurons but decreases those of Thy1- neurons in amygdala. **(A)** Representative traces of the firing pattern of a Thy1+ neuron in control mice in response to current injections (250 pA, 1 s) when the slice was perfused with ACSF, THIP, and THIP+ PTX successively. **(B)** Summary plots of the spikes number as a function of current strength, as in **(A)**. **(C)** Comparisons of the curve slope in **(B)**. **(D)** Same illustrations as in **(A)** except the data were obtained from CSDS mice. **(E)** Summary plots of the spikes number as a function of current strength, as in **(D)**. **(F)** Comparisons of the curve slope in **(E)**. **(G)** Same as in **(A)** except that the data were collected from Thy1- neurons. **(H)** Summary plots of the spikes number as a function of current strength, as in **(G)**. **(I)** Comparisons of the curve slope in **(H)**. **(J)** Same plotting of neuronal firing as in **(D)** except the data were obtained from Thy1- neuron. **(K)** Summary plots of the spikes number as a function of current strength, as in **(J)**. **(L)** Comparisons of the curve slope in **(K)**. *p < 0.05, **p < 0.01, ***p < 0.001 vs. THIP + PTX group/THIP group,^ ##^*p* < 0.01, ^###^*p* < 0.001 vs. ACSF group by THIP group. Pooled data are presented as mean ± SEM.

Data were analyzed using Student *t* test (Figure 1 and Supplementary Figure S1) or one-way (Figure 7 and Supplementary Figure S4) or two-way (Figures 2–7 and Supplementary Figures S2, S3) analysis of variance (ANOVA) with or without repeated measures, followed by *post hoc* test with Bonferroni correction. As for the cumulative distribution of frequency, amplitude, rise time, and decay time of sIPSCs (Figures 3, 6 and Supplementary Figures S2, S3), these data were analyzed by Kolmogorov–Smirnov test. All statistical analyses were performed using GraphPad Prism (GraphPad Software Inc., San Diego, CA, United States). Data are presented as means ± SEM. The threshold for statistical significance was *p* < 0.05.

## Results

### CSDS Increases the Anxiety-Like Behavior in Mice

We first used OFT and EPM to test whether the CSDS used in the present study had anxiogenic effects (Figure 1A). Relative to the unstressed controls, the CSDS mice spent less time in (unpaired *t*-test, *p* < 0.001; control, *n* = 10; CSDS, *n* = 10; Figures 1B,C) and had less entries to (unpaired *t*-test, *p* < 0.001; Figures 1B,D) the center area of open field chamber during OFT. The stressed mice also spent less time in the open arms (unpaired *t*-test, *p* < 0.001; Figures 1E,F) and had less entries to these arms (unpaired *t*-test, *p* < 0.001; Figures 1E,G) of EPM. Besides, no significant differences were observed in the EPM closed arms entries (unpaired *t*-test, *p* = 0.2499; Supplementary Figure S1A), locomotion in OFT (unpaired *t*-test, *p* = 0.7482; Supplementary Figure S1B), and EPM (unpaired *t* test, *p* = 0.8648; Supplementary Figure S1C) of these two groups. These data suggest that CSDS significantly increases the anxiety-like behavior in mice.

### CSDS Oppositely Regulates Tonic Inhibition in Thy1+ and Thy1- Neurons

Next, we explored the influence of CSDS on the GABAergic currents in Thy1+ and Thy1- PNs in basal amygdala (Figure 2A). Our recent work has shown that chronic stress readily suppresses the tonic GABA_A_R currents in the PNs of lateral amygdala (LA), but has little influence on the phasic GABA_A_R currents (Liu et al., 2014). We first examined the effects of CSDS on the tonic currents induced by THIP, a super agonist of GABA_A_R, in Thy1+ and Thy1- PNs, which could be readily differentiated by the presence or absence of fluorescent labeling (Figures 2B,C). As shown in Figures 2D,E, two-way ANOVA revealed significant effect of Thy1 expression × CSDS interaction [Thy1 expression: *F*_(__1_, _59__)_ = 0.0928, *p* = 0.7617; CSDS: *F*_(__1_, _59__)_ = 0.1645, *p* = 0.6866; interaction: *F*_(__1_, _59__)_ = 14.63, *p* < 0.001; for Thy1+ neurons, *n* = 16 neurons/8 control mice, *n* = 15 neurons/8 CSDS mice; for Thy1- neurons, *n* = 15 neurons/8 control mice, *n* = 17 neurons/8 CSDS mice]. *Post-hoc* analysis revealed that in unstressed mice the tonic currents were far larger in Thy1- PNs than their Thy1+ counterparts (Thy1+ vs. Thy1-, *p* = 0.0328). However, in CSDS mice, the tonic inhibition in Thy1+ PNs was stronger than that in Thy1- PNs (Thy1+ vs. Thy1-, *p* = 0.0093), suggesting CSDS has opposite effects on the tonic inhibition in the two populations. While markedly increasing the tonic currents in Thy1+ cells (control vs. CSDS, *p* = 0.0086), it significantly decreased those in Thy1- ones (control vs. CSDS, *p* = 0.0359).

To further confirm the above findings, we then resumed to test the effect of GABA, the endogenous agonist of GABA_A_R, on the tonic inhibition onto the two neuronal populations. Similar to the results of THIP, two-way ANOVA revealed significant effect of Thy1 expression × CSDS interaction [Thy1 expression: *F*_(__1_, _41__)_ = 13.00, *p* < 0.001; CSDS: *F*_(__1_, _41__)_ = 0.3664, *p* = 0.5483; interaction: *F*_(__1_, _41__)_ = 64.62, *p* < 0.001; for Thy1+ neurons, *n* = 10 neurons/5 control mice, *n* = 13 neurons/6 CSDS mice; for Thy1- neurons, *n* = 10 neurons/5 control mice, *n* = 12 neurons/6 CSDS mice; Figures 2F,G]. Relative to the Thy1+ PNs, GABA also evoked stronger tonic inhibitory currents in Thy1- PNs in control mice (Thy1+ vs. Thy1-, *p* = 0.0098), subsequent to CSDS, but weaker currents in CSDS mice (Thy1+ vs. Thy1-, *p* < 0.001). CSDS increased GABA-mediated tonic currents in Thy1+ PNs (control vs. CSDS, *p* < 0.001) but decreased those in Thy1- neurons (control vs. CSDS, *p* < 0.001). Altogether, these data indicate the tonic inhibition is differently expressed in the two PN populations and undergoes opposite regulation by CSDS.

### CSDS Affects Neither Phasic GABA_A_R Currents nor GABA_B_R Currents in Thy1+ and Thy1- Neurons

In addition to activating extrasynaptic GABA_A_Rs to generate tonic inhibition, GABA could also activate the synaptic GABA_A_Rs to produce the phasic inhibitory currents. We next explored the effect of CSDS on the sIPSCs by synaptic GABA_A_Rs in the two populations. As shown in Figure 3 and Supplementary Figure S2, both Thy1 expression and CSDS had no main effects on the mean frequencies [Thy1 expression: *F*_(__1_, _36__)_ = 0.0374, *p* = 0.8477; CSDS: *F*_(__1_, _36__)_ = 0.0529, *p* = 0.8194; interaction: *F*_(__1_, _36__)_ = 0.1739, *p* = 0.6792; Thy1+ neurons, *n* = 11 neurons/5 control mice, *n* = 10 neurons/5 CSDS mice; Thy1- neurons, *n* = 9 neurons/5 control mice, *n* = 10 neurons/5 CSDS mice; Figures 3A–C], amplitudes [Thy1 expression: *F*_(__1_, _36__)_ = 0.2961, *p* = 0.5897; CSDS: *F*_(__1_, _36__)_ = 0.1555, *p* = 0.6956; interaction: *F*_(__1_, _36__)_ = 0.2092, *p* = 0.6501; Figures 3A,D,E], rise time [Thy1 expression: *F*_(__1_, _36__)_ = 0.0026, *p* = 0.9597; CSDS: *F*_(__1_, _36__)_ = 0.0402, *p* = 0.8421; interaction: *F*_(__1_, _36__)_ = 0.0002, *p* = 0.9902; Supplementary Figures S2A,B], or decay time [Thy1 expression: *F*_(__1_, _36__)_ = 0.0026, *p* = 0.9594; CSDS: *F*_(__1_, _36__)_ = 0.0039, *p* = 0.9506; interaction: *F*_(__1_, _36__)_ = 0.1984, *p* = 0.6587; Supplementary Figures S2C,D] of sIPSCs, arguing against a role of CSDS in tuning phasic GABA_A_R currents in BLA PNs.

In neurons, GABA could also bind to the metabolic B-type GABA receptor (GABA_B_R) to shut down the neuronal activation. We next tested whether CSDS could regulate the GABA_B_R currents in the two PN populations, as it did on the tonic GABA_A_R currents (Figure 4A). Two-way ANOVA revealed significant main effect of Thy1 expression but not CSDS on the GABA_B_R-mediated currents [Thy1 expression: *F*_(__1_, _38__)_ = 11.61, *p* = 0.0016; CSDS: *F*_(__1_, _38__)_ = 0.0871, *p* = 0.7695; interaction: *F*_(__1_, _38__)_ = 0.1723, *p* = 0.6804; for Thy1+ neurons, *n* = 10 neurons/5 control mice, *n* = 11 neurons/5 CSDS mice; for Thy1- neurons, *n* = 11 neurons/5 control mice, *n* = 10 neurons/5 CSDS mice; Figure 4B]. *Post-hoc* results showed that in control mice the GABA_B_R currents were more prominent in Thy1- than those in Thy1+ PNs (Thy1+ vs. Thy1-, *p* = 0.0204); however, in CSDS mice, there was a significant tendency that Thy1+ neurons exhibited a higher amplitude of GABA_B_R currents (Thy1+ vs. Thy1-, *p* = 0.0819).

### Chronic Corticosterone Administration Mimics the Effects of CSDS on the Tonic and Phasic GABA_A_R Currents in Thy1+ and Thy1- PNs

Chronic stress exposure generally leads to increased secretion of glucocorticoid hormone (corticosterone in rodents), which is tightly associated with stress adversities at levels ranging from cell to behavior (Sapolsky, 2015). Chronic CORT treatment has been shown to imitate various cellular and behavioral phenotypes by chronic stress (Moda-Sava et al., 2019). To examine whether CORT was sufficient to cause distinct effect on the tonic inhibition in BLA PNs, we chronically administered the mice with CORT for a continuum of 10 days by adding CORT to the drinking water (Figure 5A). Similar to the effect of CSDS, two-way ANOVA revealed that chronic CORT administration also oppositely affected the tonic inhibition in Thy1+ and Thy1- PNs [Thy1 expression: *F*_(__1_, _34__)_ = 0.0330, *p* = 0.8569; CORT administration: *F*_(__1_, _34__)_ = 0.1239, *p* = 0.7270; interaction: *F*_(__1_, _34__)_ = 19.98, *p* < 0.001; for Thy1+ neurons, *n* = 10 neurons/5 vehicle mice, *n* = 9 neurons/5 CORT mice; for Thy1- neurons, *n* = 10 neurons/5 vehicle mice, *n* = 9 neurons/5 CORT mice; Figures 5B,C]. It up-regulated the tonic GABA_A_R currents in Thy1+ neurons (vehicle vs. CORT, *p* = 0.0034), but down-regulated that in Thy1- neurons (vehicle vs. CORT, *p* = 0.0126). The phasic GABA_A_Rs current, as measured by sIPSCs, remained unaltered in both populations following chronic CORT administration [frequency, Thy1 expression: *F*_(__1_, _34__)_ = 0.0002, *p* = 0.9877; CORT administration: *F*_(__1_, _34__)_ = 0.0414, *p* = 0.8401; interaction: *F*_(__1_, _34__)_ = 0.2071, *p* = 0.6520 (Figures 6A–C); amplitude, Thy1 expression: *F*_(__1_, _34__)_ = 0.2843, *p* = 0.5974; CORT administration: *F*_(__1_, _34__)_ = 0.1563, *p* = 0.6951; interaction: *F*_(__1_, _34__)_ = 0.2049, *p* = 0.6537 (Figures 6A,D,E); rise time, Thy1 expression: *F*_(__1_, _34__)_ = 0.2862, *p* = 0.5962; CORT administration: *F*_(__1_, _34__)_ = 0.0007, *p* = 0.0170; interaction: *F*_(__1_, _34__)_ = 0.2545, *p* = 0.6172 (Supplementary Figures S3A,B); decay time, Thy1 expression: *F*_(__1_, _34__)_ = 0.0881, *p* = 0.7684; CORT administration: *F*_(__1_, _34__)_ = 0.2121, *p* = 0.6480; interaction: *F*_(__1_, _34__)_ = 0.2536, *p* = 0.6178 (Supplementary Figures S3C,D)]. These results indicate that the increased CORT secretion may contribute to the opposite regulation of CSDS on the tonic inhibition in both Thy1+ and Thy1- neurons in BLA.

### CSDS Oppositely Regulates the GABAergic Suppression of Neuronal Excitability in Thy1+ and Thy1- Neurons

Persistent activation of extrasynaptic GABA_A_R is known to suppress neuronal activity (Trujeque-Ramos et al., 2018). Given the robust but opposite effects of CSDS on the tonic GABA_A_R currents in Thy1- and Thy1+ PNs in BLA, we speculated that CSDS might differently affect GABAergic suppression of neuronal activation in these two populations. To test this, we first injected the depolarizing current pulses with increasing intensity to the recorded neurons to evoke action potentials. Slices were then sequentially perfused with ACSF, THIP, and THIP + PTX. Somewhat surprisingly, we found that both THIP and THIP + PTX had little effect on the spike number in Thy1+ neurons from control mice [pharmacological manipulation: *F*_(__2_, _20__)_ = 1.229, *p* = 0.3137; depolarization steps: *F*_(__4_, _40__)_ = 48.13, *p* < 0.001; interaction: *F*_(__8_, _80__)_ = 1.375, *p* = 0.2204, *n* = 11 neurons/5 mice; Figures 7A,B]. However, in CSDS mice, THIP markedly decreased the spike number in these cells, an effect that was completely blocked by subsequent PTX application [pharmacological manipulation: *F*_(__2_, _16__)_ = 8.756, *p* = 0.0027; depolarization steps: *F*_(__4_, _32__)_ = 25.89, *p* < 0.001; interaction: *F*_(__8_, _64__)_ = 2.937, *p* = 0.0074, *n* = 9 neurons/5 mice; Figures 7D,E]. By contrast, THIP markedly decreased the spike number in Thy1- PNs from control mice [pharmacological manipulation: *F*_(__2_, _20__)_ = 9.191, *p* = 0.0015; depolarization steps: *F*_(__4_, _40__)_ = 43.65, *p* < 0.001; interaction: *F*_(__8_, _80__)_ = 4.305, *p* < 0.001, *n* = 11 neurons/5 mice; Figures 7G,H], but had no effect in those from CSDS mice [pharmacological manipulation: *F*_(__2_, _18__)_ = 1.120, *p* = 0.3479; depolarization steps: *F*_(__4_, _36__)_ = 60.93, *p* < 0.001; interaction: *F*_(__8_, _72__)_ = 0.2762, *p* = 0.9718, *n* = 10 neurons/5 mice; Figures 7J,K]. On the other hand, when we measured the THIP effect on the slopes of fitted curves plotting the spike number against the current strength, similar results were observed. THIP had little effect on the slope in Thy1+ cells from control mice [*F*_(__2_, _20__)_ = 2.835, *p* = 0.0824; Figure 7C] but decreased it in those from CSDS mice [*F*_(__2_, _16__)_ = 6.641, *p* = 0.0079; Figure 7F]. By contrast, for Thy1- cells, THIP decreased the slope from control mice [*F*_(__2_, _20__)_ = 9.686, *p* = 0.0011; Figure 7I] while effected little in CSDS mice [*F*_(__2_, _18__)_ = 0.0832, *p* = 0.9206; Figure 7L].

We also used an alternative approach to elicit neuronal firing by injecting a ramped current (0–250 pA, 1 s) into the recorded cells. One-way ANOVA revealed that the effects of THIP on the spike number in the two populations from control and CSDS mice were in good accordance with the effects observed when current pulses were used [Thy1+ neurons from control mice, *F*_(__2_, _20__)_ = 3.023, *p* = 0.0712; Supplementary Figures S4A,B; Thy1+ neurons from CSDS mice, *F*_(__2_, _16__)_ = 7.825, *p* = 0.0043; Supplementary Figures S4C,D; Thy1- neurons from control mice, *F*_(__2_, _20__)_ = 10.40, *p* < 0.001; Supplementary Figures S4E,F; Thy1- neurons from CSDS mice, *F*_(__2_, _18__)_ = 2.331, *p* = 0.1258; Supplementary Figures S4G,H].

Altogether, these findings strongly suggest that under physiological conditions the tonic GABA_A_R currents differently affect the activity of Thy1+ vs. Thy1- PNs in BLA. Chronic social defeat stress, on the other hand, oppositely affects the effects of tonic GABAergic currents in the two populations.

## Discussion

Here, we showed that CSDS drastically increased the tonic GABA_A_R currents in Thy1+ neurons, but decreased the currents in Thy1- ones in BLA. It had no effect on the phasic GABA_A_R currents or GABA_B_R currents in both populations. Consequently, the GABAergic suppression of neuronal excitability in Thy1+ and in Thy1- neurons was augmented and removed by CSDS, respectively. The opposite regulation of CSDS on the tonic currents in these two populations could be readily mimicked by chronic CORT administration.

Tonic inhibition, which is mediated by extrasynaptically located GABA_A_Rs, has been extensively observed in many brain regions including cerebellum, hippocampus, amygdala, and neocortex (Brickley and Mody, 2012). Accumulating evidence indicates that the tonic inhibition in amygdala plays a key role in the inhibitory control of amygdala activity (Marowsky et al., 2012; Martin et al., 2014; Liu et al., 2017; Meis et al., 2018). Surprisingly, our results showed that the expression of tonic inhibition in BLA PNs varied drastically with the presence or absence of Thy1, and the tonic GABA_A_R currents in Thy1+ neurons were far lower than those in Thy1- PNs under normal physiological conditions (control mice). Two possible explanations may underlie this. One is that the tonic inhibition of these two groups of PNs in the amygdala is independent of Thy1 expression. The expression pattern of GABA_A_Rs-mediated tonic currents is coincidently overlapped with that of Thy1. Notably, it has been reported that the nucleus accumbens (NAc) and central medial amygdala projectors within the BLA may differentially express tonic inhibition. For example, the NAc projecting neurons express much less Gabrd mRNA, which encodes δ subunit of GABA_A_Rs that mediates tonic inhibition, than the CeM projecting ones (Namburi et al., 2015). The BLA Thy1+ neurons were primarily terminated in the NAc (Porrero et al., 2010; McCullough et al., 2016), whereas their axons are rarely found in CeM (McCullough et al., 2016). The other one may be that the Thy1 acts in gating the expression of tonic inhibition. Indeed, Nosten-Bertrand et al. (1996) have demonstrated a potential link between Thy1 and GABAergic signals in hippocampus. The Thy1 null mice exhibited impaired LTP in dentate gyrus of the hippocampus, and such impairment could be rescued by bicuculline, an antagonist of GABA_A_R (Nosten-Bertrand et al., 1996). However, because many subunits of GABA_A_Rs such as δ, α3, and α5 have been shown to be involved in the tonic inhibition in amygdala (Marowsky et al., 2012; Martin et al., 2014; Liu et al., 2017; Meis et al., 2018), how Thy1 affects the expression and function of these subunits remains to be unknowns and awaits further investigation.

Notably, tonic inhibition has been shown to be regulated by chronic stress. For example, our previous work showed that chronic restraint stress reduces tonic inhibition in BLA projection neurons (Liu et al., 2014). However, these results are obtained from the BLA PNs as a whole, whether the tonic inhibition in individual BLA PNs is differentially regulated by chronic stress remains unknown. Here we observed that CSDS up- and down-regulated the tonic inhibitory currents in Thy1+ and Thy1- neurons, respectively, suggesting the tonic inhibition in the two populations is differentially regulated by CSDS. This may result from the fact that CSDS induces different alteration of the expression of GABA_A_Rs subunits that mediates tonic inhibition in Thy1+ /Thy1- neurons. Of note, it has been documented that chronic stress can specifically alter the expressions of distinct GABA_A_R subunits even in the same brain area (Gunn et al., 2011; Maguire, 2014). For example, in paraventricular nuclei (PVNs), chronic unpredictable stress decreases the expression of δ subunit but increases that of α5 subunit, whereas it has no effect on α3 subunit expression (Verkuyl et al., 2004). These subunits-containing GABA_A_Rs have been reported in helping with formatting tonic inhibition in amygdala. However, which subunit(s) is (are) responsible for mediating CSDS-induced changes of tonic inhibition in these two neuronal populations needs to be further identified.

In sharp contrast to the tonic inhibition, the phasic inhibition mediated by synaptic GABA_A_Rs was not affected by CSDS (as indicated by no changes in the amplitude, frequency, and kinetics of sIPSCs) in both Thy1+ and Thy1- neurons. These results appear to be consistent with previous studies that continuous stress exposure had little influence on phasic inhibition in BLA PNs. Jiang et al. (2009) reported that 3 consecutive days of immobilization and tail shock stress did not affect sIPSCs in amygdala. Moreover, Liu et al. (2014) also demonstrated that the sIPSC in LA PNs remained unaltered after 10 days of chronic restraint stress or chronic unpredictable stress. Not only this, we also found that the GABA_B_R currents, another important inhibitory current contributing to the inhibition in amygdala (Pan et al., 2009), are not changed after CSDS. However, studies also revealed that chronic stress exposure enables GABA_B_R internalization to down-regulate GABA_B_R current in lateral habenula (Lecca et al., 2016) and PVN (Gao et al., 2017), suggesting a possibility that chronic stress may have distinct impacts on the GABA_B_R currents in different brain regions.

A large number of studies have shown that chronic stress mediates its negative effects through elevating CORT levels (Herman, 2013; Sapolsky, 2015). In accordance with this, we showed that chronic feeding with CORT also mimicked the effect of CSDS on the tonic GABA_A_R currents in distinct neuron populations, implying that CORT may participate in CSDS-induced differential regulation of GABA_B_R currents. However, how CORT differentially regulates the tonic GABA_A_R currents in Thy1+ and Thy1- neurons remains elusive. Our previous study has demonstrated that chronic restraint stress reduces the tonic inhibition in BLA PNs as consequence of activation of glucocorticoid receptor (GR), which indicates a pivotal role of GR in modulating tonic inhibition in amygdala (Liu et al., 2014). Indeed, not all PNs in amygdala express GR, and the function of GR varies in different types of neurons (Groeneweg et al., 2011; Gunduz-Cinar et al., 2019). Thus, it is likely that the heterogeneity of the expression and functionality of GR in amygdala may contribute to the differential regulation of tonic inhibition in Thy1+ and Thy1- neurons. Moreover, accumulating evidence reveals that chronic CORT exposure has diverse effects on different subunits of GABA_A_R (Orchinik et al., 1995, 2001; Cullinan and Wolfe, 2000). The unique receptor composition of GABA_A_R in distinct BLA PNs may also account for the differential regulation of GABA_A_R-mediated tonic inhibition by CORT. On the other hand, the secretion of many kinds of neurosteroids, such as allopregnanolone (THP), can be elevated by stress (Gunn et al., 2011). These neurosteroids can directly interact with and modulate membrane receptors, such as the GABA_B_R (Reddy and Estes, 2016; Olsen, 2018). For example, the fluctuation of THP has been implicated in altering the potency and expression of extrasynaptic located GABA_A_Rs, especially the δ subunit–containing GABA_A_Rs (Maguire, 2014). In the hippocampal CA1 region of female rodents, a relative short-term (48 h) THP administration caused an elevated expression of δ subunit of GABA_A_Rs, and increased THIP induced tonic currents consequently (Shen et al., 2005). Nevertheless, prolonged THP exposure in pubertal female mice blocked the expression of GABA_A_Rs at a composition of α4β2δ (Shen et al., 2007). Moreover, THP functions oppositely on tonic inhibition in different compositions of GABA_A_Rs; it decreases outward current of α4β2δ GABA_A_Rs and increases that of α4β2γ2, α4β3δ, α4β2, and α1β2δ GABA_A_Rs, whereas it has no effect on α5β2/3γ GABA_A_Rs (Shen et al., 2007), suggesting a paradoxical role of THP in affecting tonic inhibition. On this account, THP may serve on the differential modulation of the tonic inhibition in Thy1+ and Thy1- neurons in the BLA by chronic stress.

Persistent and excessive activation of amygdala following chronic stress contributes to the pathogenesis of stress-related mental disorders (McLaughlin et al., 2009). Consistent with the different changes of tonic inhibition in Thy1+ and Thy1- neurons, we found that CSDS augmented GABAergic suppression of neuronal excitability in Thy1+ neurons but impaired that in Thy1- neurons. Considering the charge transfer mediated by extrasynaptic GABA_A_R is substantially larger than that of synaptic GABA_A_R (Botta et al., 2015) and the phasic inhibition in both neuron populations remains unaltered subsequent to CSDS, the altered GABAergic suppression of neuronal excitability is most likely mediated by tonic inhibition. Previous studies have shown that activation of Thy1+ neurons in BLA (“fear-off” neurons) inhibits fear expression and promotes fear extinction (Jasnow et al., 2013; McCullough et al., 2016). Notably, the Thy1- ones are also activated during fear retrieval (McCullough et al., 2016). We speculate that CSDS increased the neuronal excitability in Thy1+ BLA neurons but reduced that of Thy1- neurons, which is potential to cause excessive fear. However, optogenetically activating Thy1+ neurons had little anxiogenic effects (Jasnow et al., 2013). This seems inconsistent with our findings; one possible explanation could be that stress-induced anxiety-like behavior may be mediated by Thy1- neurons rather than Thy1+ ones, or these two neuron groups work synergistically. Actually, the function of Thy1+ and Thy1- neurons is closely related to their projection patterns. As we discussed earlier, Thy1+ cells in BLA were reported as preferentially projecting to the brain area where positive valence is mediated such as NAc and bed nucleus of the stria terminalis (McCullough et al., 2016); the increased suppression of Thy1+ neurons by GABA_A_Rs following CSDS may actively contribute to its negative influences on emotion and behavior. On the other hand, Thy1+ neurons rarely project to the CeM (probably Thy1- neurons) (Jasnow et al., 2013), and activation of BLA PNs projecting to CeM contributes to aversive behaviors (Namburi et al., 2015). Moreover, BLA neurons projecting to ventral hippocampus are dramatically activated by chronic stress and are responsible for stress-induced increase of anxiety-like behavior (Zhang J.Y. et al., 2019; Zhang W.H. et al., 2019). Thus, the decreased suppression of Thy1- neurons by GABA_A_Rs following CSDS may also participate in stress-related psychiatric diseases, while the exact neuron type needs to be identified in future study.

Taken together, our results show that CSDS differentially regulates the tonic GABA_A_R currents in Thy1+ and Thy1- neurons in BLA. In parallel with this finding, the GABAergic control over neuronal firing is also oppositely modulated by CSDS. Despite this, several important questions remain open. For example, what are the specific molecular mechanisms underlying such differential regulation? What is the functional role of the divergent modulation of tonic inhibition in stress-related emotional disorders? Addressing these issues will help to expand our understanding of the molecular and circuit mechanisms of the stress-mediated amygdala disinhibition and provide potential targets for prevention and treatment of stress-related disorders.

## Data Availability Statement

The raw data supporting the conclusions of this article will be made available by the authors, without undue reservation, to any qualified researcher.

## Ethics Statement

The animal study was reviewed and approved by the Institutional Animal Care and Use Committee of Nanchang University.

## AuthOr Contributions

B-XP and W-HZ conceived and designed the project. H-QP, W-HZ, and C-ZL performed most experiments and analysis of electrophysiology data. YH performed immunofluorescence experiments. Z-MX, XQ, and W-ZL made the mice models of chronic social defeat and chronic CORT administration. NW, J-XZ, and X-XL performed behavior test. B-XP, H-QP, and W-HZ wrote the manuscript. All authors read and approved the final manuscript.

## Conflict of Interest

The authors declare that the research was conducted in the absence of any commercial or financial relationships that could be construed as a potential conflict of interest.

## Supplementary Material

FIGURE S1 There’s no differences on the entries to the closed arms of EPM and locomotor activity between control and CSDS mice. **(A)** Closed arms entries. **(B)** Total distance traveled during OFT. **(C)** Total distance traveled in EPM.

FIGURE S2 No significant differences on rise time and decay time of sIPSCs by CSDS. **(A)** Average of sIPSC rise time. **(B)** Cumulative probability of rise time of sIPSC. **(C)** Average of sIPSC decay time. **(D)** Cumulative probability of decay time of sIPSC.

FIGURE S3 No significant differences on rise time and decay time of sIPSCs by CORT administration. **(A)** Average of sIPSC rise time. **(B)** Cumulative probability of rise time of sIPSC. **(C)** Average of sIPSC decay time. **(D)** Cumulative probability of decay time of sIPSC.

FIGURE S4 CSDS differentially modulates tonic inhibitory control over neural firing of Thy1+ and Thy1- neurons in amygdala. **(A,C)** Representative traces of the firing pattern of Thy1+ neurons from control **(A)** and CSDS mice **(C)** in response to a ramp current injection (0–250 pA, 1 s). **(B,D)** Summary plots of the spike number in **(A)** and **(C)**, respectively. **(E,G)** Representative traces of the firing pattern of Thy1- neurons from control **(E)** and CSDS mice **(G)**. **(F,H)** Summary plots of the spike number in **(E,G)**, respectively. **p* < 0.05, ***p* < 0.01, ****p* < 0.001. Pooled data are presented as mean ± SEM.
